# Expression of PD-L1 and other immunotherapeutic targets in thymic epithelial tumors

**DOI:** 10.1371/journal.pone.0182665

**Published:** 2017-08-03

**Authors:** Kathryn C. Arbour, Jarushka Naidoo, Keith E. Steele, Ai Ni, Andre L. Moreira, Natasha Rekhtman, Paul B. Robbins, Joyson Karakunnel, Andreas Rimner, James Huang, Gregory J. Riely, Matthew D. Hellmann

**Affiliations:** 1 Thoracic Oncology Service, Division of Solid Tumor Oncology, Department of Medicine, Memorial Sloan Kettering Cancer Center, New York, New York, United States of America; 2 MedImmune LLC, Gaithersburg, Maryland, United States of America; 3 Department of Biostatistics, Memorial Sloan Kettering Cancer Center, New York, New York, United States of America; 4 Department of Pathology, Memorial Sloan Kettering Cancer Center, New York, New York, United States of America; 5 Department of Radiation Oncology, Memorial Sloan Kettering Cancer Center, New York, New York, United States of America; 6 Thoracic Service, Department of Surgery, Memorial Sloan Kettering Cancer Center, New York, New York, United States of America; 7 Department of Medicine, Weill Cornell Medical College, New York, New York, United States of America; University of South Alabama Mitchell Cancer Institute, UNITED STATES

## Abstract

**Introduction:**

The thymus is a critical organ for the development of the adaptive immune system and thymic epithelial tumors (TETs; thymomas and thymic carcinomas) are often associated with auto-immune paraneoplastic conditions. However, the immunobiology of TETs is not well described. An evaluation of the tumor microenvironment, with particular focus on expression of immunotherapeutic targets, may facilitate and prioritize development of immunotherapy strategies for patients with TETs.

**Methods:**

Tumor tissues from 23 patients with WHO Type B2/B3 thymoma (n = 12) and thymic carcinoma (n = 11) were identified and clinical outcomes were annotated. The expression of membranous PD-L1 on tumor cells, CD3+ and CD8+ tumor infiltrating lymphocytes (TILs), co-stimulatory (CD137, GITR, ICOS), and co-inhibitory immune checkpoint molecules (PD-1, CTLA-4, TIM-3) were assessed semi-quantitatively using immunohistochemistry.

**Results:**

PD-L1 positivity (≥ 25% of tumor membrane expression) was frequent in TETs (15/23, 65%), more common in thymomas compared to thymic carcinomas (p<0.01), and was associated with longer overall survival (p = 0.02). TIM-3 and GITR were expressed in all TETs, including 18/23 and 12/23 with at least moderate/high expression, respectively. Moderate/high CD137 expression correlated with CD8+ (p = 0.01) and moderate/high GITR expression co-associated with PD-1 (p = 0.043).

**Conclusions:**

TETs are characterized by frequent PD-L1 expression and PD-L1 is associated with improved survival, suggesting PD-L1 signaling may be biologically important in TETs. Robust expression of markers of immune activation and immunotherapeutic target molecules in TETs emphasizes the potential for development of anti-PD-1/PD-L1 therapies.

## Introduction

The thymus is a critical lymphoid organ in the development of the adaptive immune system during childhood, but through gradual involution becomes largely atrophic in adults. In relatively rare cases, residual epithelial tissue can become neoplastic. Thymic epithelial tumors (TETs) are classified as thymomas (WHO types A, AB, B1, B2, and B3) or thymic carcinomas (WHO type C) based on the morphology of epithelial tumor cells and proportion of lymphocytic involvement.[1] While thymomas are frequently associated with a wide range of autoimmune paraneoplastic disorders[2] these disorders are rarely seen in patients with thymic carcinoma.[3]

Standard initial treatment regimens for patients with advanced TETs include those with anthracycline and/or platinum[4–6]. After progression of disease, targeted therapies have been evaluated but are associated with modest response rates.[7–9] Given the importance of the thymus in the development of the adaptive immune system, there has been interest in examining the potential role of immune checkpoint therapies in TETs. However, as these therapies can also be associated with potentially severe auto-immune toxicity, there we sought to assess the immune microenvironment prior to more empiric approaches to evaluating these therapies which may enable more efficient drug development.

We hypothesized that evaluation of the tumor microenvironment of TETs, with a particular focus on targets with therapeutic agents currently in development, may reveal subgroups of distinct immunophenotypes and inform the safe and effective development of relevant immunotherapy agents for patients with TETs. We examined this hypothesis by evaluating the presence of PD-L1 using a clinically validated antibody, assessing for membranous staining. In addition to PD-L1, other co-inhibitory and co-stimulatory molecules were evaluated, as well as quantifying subsets of tumor-infiltrating lymphocytes (TILs). We also sought to characterize the interplay between these multiple markers and T cell subsets including CD3 and CD8 expression. This analysis was performed in a series of TETs with histologic features[10] associated with high risk for recurrence and poor prognosis thereby focusing on a group most likely to require systemic therapy, and be enrolled in future clinical trials with immunotherapeutic agents.

## Methods

Following approval by the Memorial Sloan Kettering Cancer Center institutional review board (IRB), pathologic specimens of high grade TETs (WHO Type B2 and B3 thymoma and thymic carcinoma) from patients treated at Memorial Sloan Kettering Cancer Center were identified. Diagnosis was based on WHO classification (2004 edition) and was confirmed by a thoracic pathologist (A.M., N.R.).

Sections were stained by immunohistochemistry (IHC) for PD-L1 (E1L3N, Cell Signaling Technology, Danvers, MA). Additional IHC was performed to demonstrate tumor infiltrating lymphocytes (TILs) expressing T-cell markers CD3 (clone 2GV6) and CD8 (clone SP57), co-stimulatory check point molecules CD137 (clone BBK2), glucocorticoid-induced TNFR-related protein (GITR, clone HPA008025), and inducible t-cell co-stimulator (ICOS, clone FL199) as well as co-inhibitory immune checkpoint molecules PD-1 (clone NAT105), CTLA-4 (goat polyclonal), and T-cell immunoglobulin and mucin-domain containing-3 (TIM-3, goat polyclonal). All IHC was performed on either Ventana (Ventana Medical Systems, Tucson, AZ) or DAKO (DAKO USA, Santa Clara, CA) automated instruments.

**Fig 1:**
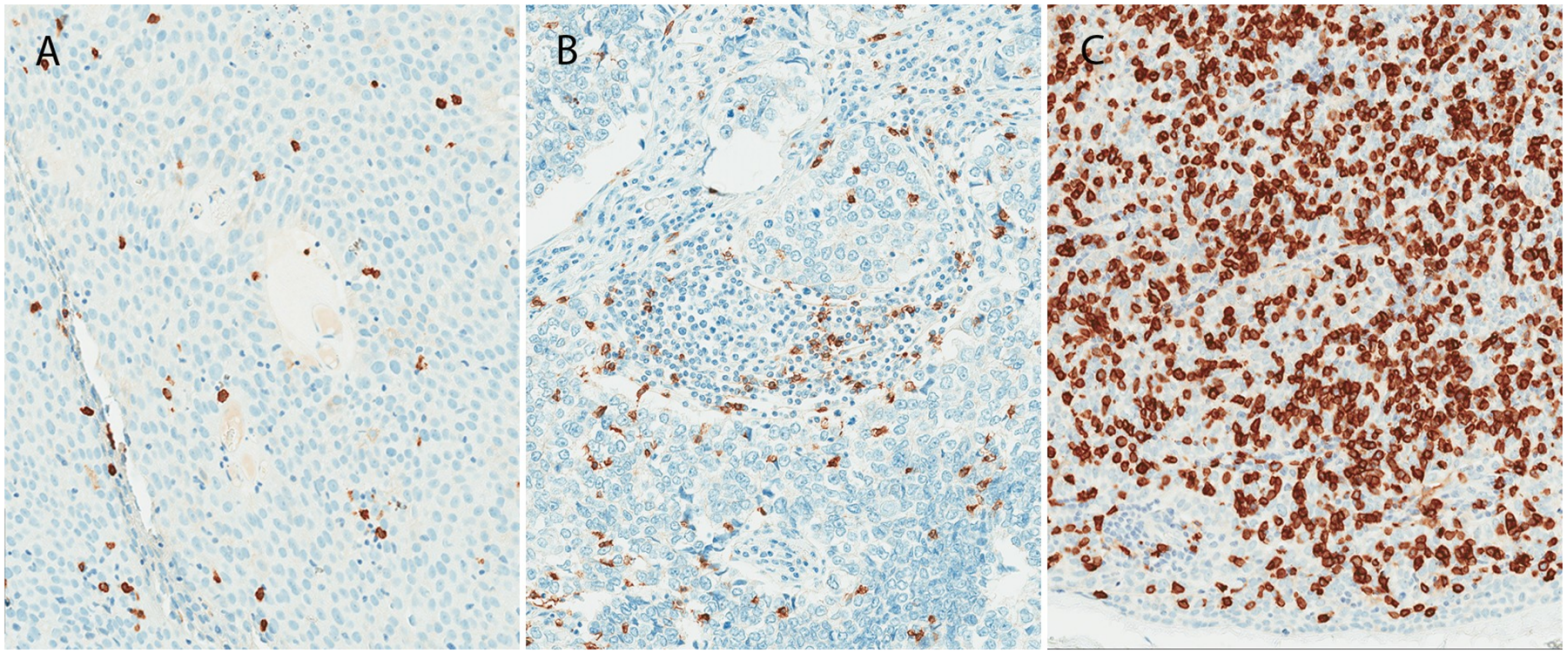
CD8 semiquantitative scoring scheme: Scoring for each specimen took into account the heterogeneity of TIL densities throughout the entire section of each tumor, here showing representative images of 1+ (A), 2+ (B) and 3+ (C) scores for CD8 representing numbers of immuno-labeled TILs per 40X microscopic field in the range of 1–30, 31–100, and >100, respectively.

PD-L1 expression was scored by a pathologist (K.S.) and quantified in two ways: 1) M-score, in which the percentage of tumor cells with membranous expression at any intensity was determined[11] and 2) H-score, in which the proportion of cells with membranous PD-L1 expression is weighted by the intensity of expression (i.e. cells with no expression x 0 + cells with low expression x 1 + cells with moderate expression x 2 + cells with high expression x 3, with a possible score range of 0–300). Based on reported clinical trials of patients treated with durvalumab in which PD-L1 expression ≥ 25% has been associated with improved outcomes,[12],[13] we pre-specified the definition of patients as PD-L1 “positive” as those with M-score ≥ 25%. For TIL markers, slides were semi-quantitatively scored by the pathologist as 0 (no labeled cells), 1+, 2+, 3+. For each marker, an individual scheme was generated to represent the range of labeled immune cells in all samples (low, medium, high) for that particular marker. Where the score for each specimen took into account the heterogeneity of TIL infiltrates throughout each tumor section, the numbers of immuno-labeled TILs per 40X microscopic field typical of 1+, 2+ or 3+ scores respectively for each marker is as follows: CD3–1-50/51-120/>120; CD8–1-30/31-100/>100; CD137–1-4/5-12/>12; CTLA-4–1-5/6-20/>20; GITR– 1-5/6-20/>20; ICOS– 1-10/11-45/>45; PD1–1-5/6-25/>25; TIM-3–1-5/6-20/>20. This scheme is illustrated for CD8 (Fig 1). Immunolabeled cells in or near the invasive margin were not counted, in order to avoid potentially interpreting resident immune cells (e.g. thymic lymphocytes) as TILs. Testing was performed on one sample per patient.

Associations between clinicopathologic factors, survival, immune checkpoint molecule expression, and TIL populations were described. Overall survival following initial diagnosis was estimated using Kaplan–Meier methodology and compared between groups by log-rank test. Association between categorical variables was assessed by Fisher’s exact test.

## Results

The clinical and pathologic features of patients with TETs and their associated tumors are summarized in Table 1 with individual details outlined in S1 Table. Most thymomas (9/12) were WHO Type B3. The majority of patients presented with Masaoka Stage III, IVA, or IVB TETs at the time of initial diagnosis (17/23). 2 patients (both with a diagnosis of thymoma), had a history of myasthenia gravis.

**Table 1:** Patient characteristics.

Variable	N	%
**Total**	23	
**Age**		
≥65	9	39
<65	14	61
**Gender**		
Male	13	57
Female	10	10
**Smoking Status**		
Never	13	57
Former/Current	10	43
**KPS**		
>80	14	61
≤80	9	39
**Histology**		
Thymoma	12	52
Thymic Carcinoma	11	48
**Stage at Diagnosis**		
I/II/III	11	48
IVA/ IVB	12	52
**Pre/Post Therapy Sample**		
Pre-chemotherapy	8	35
Post-chemotherapy	15	65

Sixty-five percent of TETs (15/23, 95% CI: 43 to 84%) were PD-L1+ using a cutoff PD-L1M-score ≥ 25%. PD-L1 positivity was more common in thymomas compared with thymic carcinomas (11/12 vs. 4/11, p<0.01) as summarized in Table 2. There was no significant difference in PD-L1 positivity when comparing samples from patients according to stage, tumor size, or site of sample (primary vs metastatic). There was also no significant difference in PD-L1 positivity, CD8 or CD3 + TILs, or expression of other co-stimulatory and co-inhibitory markers when comparing samples from patients prior to chemotherapy or after chemotherapy exposure.

**Table 2:** Histologic subtype and correlation with PD-L1 expression, CD8+ T-cells and CD3+ T-cells.

	Thymoma	Thymic Carcinoma	P value
PD-L1 (M-score)*, n = 23			P = 0.009
<25%	1	7	
>25%	11	4	
**CD8+ T-cells**^#^**, n = 23**			P = 1.0
0/1	3	3	
2/3	9	8	
**CD3 T-cells**^#^**, n = 22**			P = 1.0
0/1	5	4	
2/3	6	7	

*M-score: defined as ≥25 of 100 cells with positive membranous staining of PD-L1.

^#^CD3 and CD8 positive T-cells evaluated by immunohistochemistry, where 0/1 = low positive T cell staining, 2/ 3+ = moderate-high positive T cell staining. CD3 + TIL evaluation only available in 22 samples due to technical failure in one sample.

All tumors possessed TIL (CD3 IHC3 = 8, IHC2 = 5, IHC1 = 9, total of 22 samples due to technical failure in one sample), including CD8+ TILs in all samples (IHC3 = 7, IHC2 = 10, IHC1 = 6), as outlined in Table 2. All TETs expressed TIM-3, including 18 (73%) with moderate/high TIM-3 expression; GITR was also universally expressed in the TETs, although moderate/high expression was less frequent (12/23, 52%). ICOS and CTLA-4 were commonly expressed (21/23, 91% for each), with similar rates of high/moderate expression compared to GITR (CTLA-4: 12/23, 52%, ICOS: 11/23, 48%).

**Fig 2:**
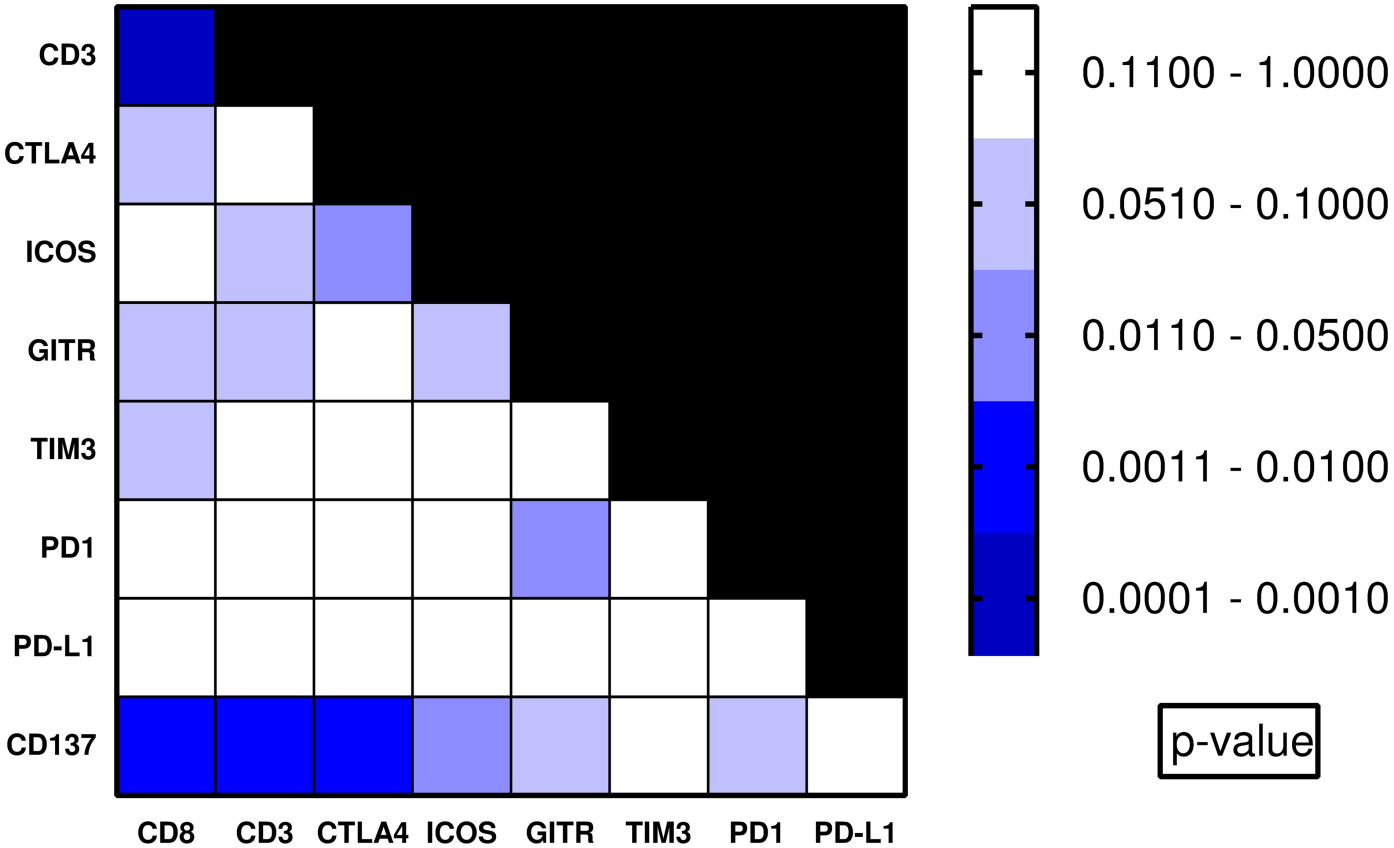
Heatmap of p-values of pairwise association analysis of co-stimulatory and co-inhibitory immune checkpoint molecules and TILs. Markers were compared as binary values, M-score: defined as ≥25 of 100 cells with positive membranous staining of PD-L1, all other markers evaluated by immunohistochemistry, where 0/1 = low positive T cell staining, 2/ 3+ = moderate-high positive T cell staining.

We next examined co-associations between therapeutic targets. Pair-wise associations between all variables were assessed, with T-cell markers considered as binary groups (high/moderate vs low/no expression) (Fig 2 and S2 Table). High/moderate CD137 expression co-associated with several markers, including CD8 (p = 0.01), CTLA-4 (p = 0.008), and ICOS (p = 0.03). High/moderate GITR expression co-associated with PD-1 (p = 0.043).

**Fig 3:**
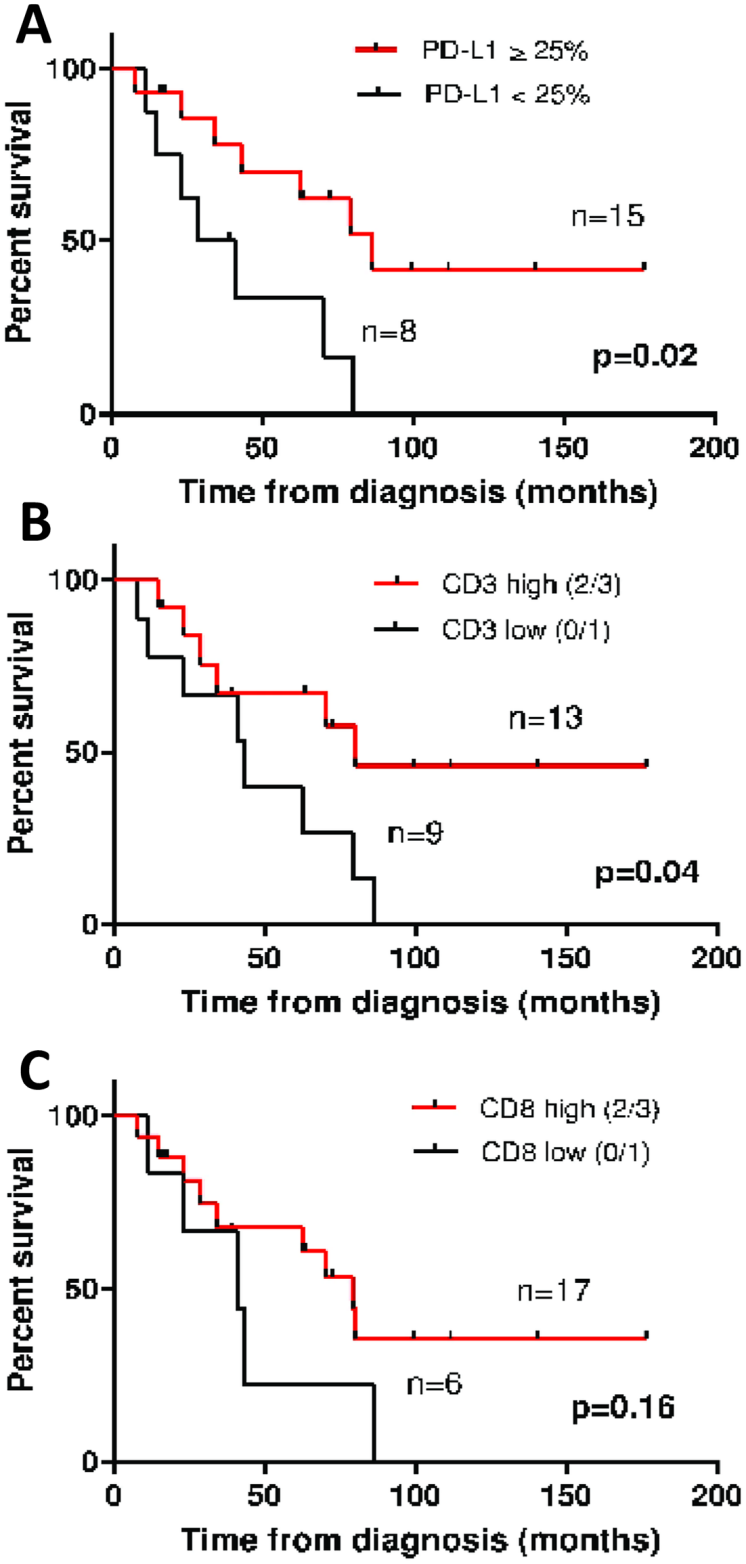
Associations of immune marker expression and TILs with overall survival from time of initial diagnosis ***A***, PD-L1 expression, where <25 deemed negative, ≥25 deemed positive. ***B***, CD3+ T-cells where 0/1 staining by IHC deemed low, 2/3 deemed high. ***C***, CD3+ T-cells where 0/1 staining by IHC deemed low, 2/3 deemed high.

The median overall survival (mOS) for all patients with TETs in our cohort was 70 months (95% CI 34–87) and was not significantly different between WHO B2/B3 thymoma and thymic carcinoma (thymoma mOS = 87 months, 95% CI 24–87, thymic carcinoma mOS = 70 months, 95%CI 29–80; p = 0.42). At time of data cutoff, 13/23 patients had died. 10/13 (77%) of patients died of thymoma or thymic carcinoma (2 patients died of other causes and the cause of 1 patient was not known). Patients with PD-L1 positive tumors had improved survival compared with patients with PD-L1 negative tumors (mOS 87 months [95% CI: 43–87] versus 29 months [95% CI: 15–70], *p* = 0.02, Fig 3A). When examining PD-L1 expression as an H score, it was not associated with OS (hazard ratio: 0.99, 95% CI 0.988–1.002, p = 0.16). The presence of moderate and high levels of CD3+ TILs (IHC 2 or 3 vs IHC 1) was also associated with an improved OS (mOS 80 months [95% CI:34–80] versus 43 months [95% CI 23–79], p = 0.04, Fig 3B). Presence of high versus low CD8+ TILs did not impact OS (Fig 3C). Individual patient data for clinicopathologic features and immunophenotyping results are provided in S3 Table.

## Discussion

Immune checkpoint inhibitors are now widely used in a variety of different cancer types[14–16], however only a subset of patients sustain clinical responses and determining the predictors of response remains a critical, ongoing effort. Accordingly, when considering new cancer indications to explore, identifying rational ways to prioritize development of the multiple immunotherapies possible to study will be critical to the development of appropriate and efficient clinical trials. In that light, we begin to profile TETs for PD-L1 expression and additional immunotherapeutic markers. We find that PD-L1 expression is frequent in TETs, particularly thymomas and those patients with PD-L1 positive TETs have distinctly improved survival compared to PD-L1 negative TETs. The finding that PD-L1 expression is associated with improved survival has been noted in other tumor types as well. For example, in patients with non-small cell lung cancers (NSCLCs), PD-L1 expression was associated with improved overall survival independent of age and stage of disease[17] and also associated with improved overall survival in early stage patients[18]. From a mechanistic perspective of the association PD-L1 and improved survival, PD-L1 expression may generally be a surrogate marker for an “inflamed” tumor microenvironment, which is consistent with an effective host response to the tumor and improved survival as a result of native anti-tumor immunity.

This association between high PD-L1 expression and improved benefit from PD-1/PD-L1 blockade in other tumor types[19] raises the possibility that anti-PD-1/PD-L1 drugs may be effective therapy for patients with TETs. Indeed, clinical trials evaluating the use of these agents in the treatment of thymic carcinoma are already underway and have demonstrated promising preliminary clinical activity.[20]

We further evaluated the tumor microenvironment for other immune co-stimulatory and co-inhibitory markers that may be targeted therapeutically, the first such evaluation in this disease. Overall, there was variability in the expression of these molecules consistent with the notion that the immune response to cancer is heterogenous.[21] However, some trends were evident. Although not associated with high PD-L1 expression, moderate to high TIM-3 expression was found in most TETs. TIM-3 has been implicated as an effective therapeutic target, which may be synergistic with anti-PD-1/PD-L1 blockade and therefore the absolute rate of high expression may be of interest for purposes of future drug development.[22] Moderate to high expression of other potentially therapeutic targets in this series were also seen for CTLA-4 (52%), GITR (52%), ICOS (48%), CD137 (54%). High expression of CD137, in particular, was frequently co-expressed with high CD8+ TILs, potentially making this relevant to explore in TETs as well. CD137 expression has recently been used to identify neoantigen-specific T cells[23] and promising data of a CD137 agonist in combination with PD-1 blockade therapy was recently presented in patients with melanoma (response rate 50% [23/46] with similar rates in patients with PD-L1 positive and negative tumors).

Prior studies have also identified PD-L1 expression in a majority of TETs. Padda et al. examined 69 TETs and found 68% of samples expressed high levels of PD-L1 using the clone 15 antibody (Sino Biological).[24] Katsuya et al. examined a larger series of 141 TETs and found high PD-L1 expression in 70% of thymic carcinoma samples but only 23% of thymoma samples stained positive for PD-L1 (Cell Signaling E1L3N clone, H-score evaluation alone)[25] and Yokoyama et al. noted high PD-L1 expression in 80% of a series of thymic carcinoma specimens (EPR1161, Abcam, H-score evaluation, included cytoplasmic expression)[26]. Direct comparisons between our study and these others are difficult due to differences in PD-L1 antibodies and criteria used to define PD-L1 positivity. In particular, it is noted that none of these studies based PD-L1 positivity on M-score, as was used in our study and is typical for current clinical trials. Furthermore, our report is distinct in using an antibody and scoring (including membranous expression only) that has been demonstrated to be consistent with assays with known predictive of therapeutic benefit with anti-PD-(L)1 therapy in clinical use and therefore has the greatest biologic and therapeutic applicability. A recent prospective report from described the consistency of the E1L3N PD-L1 assay with 22C3 and 28–8, which are now FDA approved and have important predictive validity[27] as opposed to other antibodies (e.g. Abcam EPR1161) which have no known cutpoint or predictive appreciation with response to anti-PD(L1) therapy and therefore may have limited clinical implications.

Thus far, there has been significant discrepancy regarding the prognostic impact of PD-L1 on survival[24–26,28,29]. Padda et al concluded that high PD-L1 expression associated with poorer overall survival[24] while Katsuya et al. described no survival difference associated with PD-L1 expression.[25] Meanwhile, our series suggests that high PD-L1 expression on the membrane of tumor cells is associated with improved overall survival. In contrast to the previous studies which examined TETs more broadly, we note that our series was largely comprised of patients with more aggressive histologies and advanced stage disease. Yokoyama and colleagues similarly described improved overall survival in high PD-L1 expression,[26] however this series was limited to only thymic carcinoma histology. Our series, which included patients with both thymic carcinoma and advanced stage thymoma with aggressive histologies, provides clarification on this issue. While its focus is limited to only patients with more aggressive histologies of thymic epithelial tumors (WHO Type B2, B3, and Thymic carcinoma), understanding the immunophenotype of these more aggressive tumors optimizes conclusions related to potential systemic therapies and best reflective of patients who are most likely to be treated with immunotherapies in clinical trials. These results are most reflective of the immunobiology of TETs in the setting in which immunotherapy would be clinically explored and therefore particularly valuable.

Our series is certainly limited by its size, such that larger series will be needed to more comprehensively evaluate the immunophenotypic landscape of TETs and more robustly determine associations with outcomes in a more comprehensive multivariate analysis. However, as the major intent of this analysis was to examine therapeutic targets that should be prioritized for clinical development, a modest size cohort can still provide useful insight. To this end, we hope that use of a PD-L1 assay with reliable association with clinically validated assays may help to assure its biological relevance in this disease and encourage the development of immunotherapies for patients with thymic cancers.

The development of immune checkpoint inhibitors in the treatment of TETs represents an important next step in improving outcomes in this disease. Building on studies such at this, we are hopeful that describing the presence and interconnections between immunotherapeutic targets and TILs may help to facilitate and expedite clinical trials to explore immunotherapies in TETs.

## Supporting information

S1 Table Clinicopathologic features, treatment and survival data, stratified by PD-L1 M-score. (DOCX)

S2 Table Pairwise association of co-stimulatory and co-inhibitory immune checkpoint molecules with PD-L1 positivity and TILs. (DOCX)

S3 Table Detailed clinicopathologic features and immunophenotyping data of individual patients. (XLSX)
