# Supplementary material for: Expression of PD-L1 and other immunotherapeutic targets in thymic epithelial tumors
Source: PLoS One. 2017 Aug 3;12(8):e0182665. doi: 10.1371/journal.pone.0182665 (PMC5542609; doi:10.1371/journal.pone.0182665)
Supplement: S1 Table — (DOCX) [file pone.0182665.s001.docx]

**S1 Table: Clinicopathologic Features, Treatment and Survival Data, stratified by PD-L1 M-score**

|  | **Age** | **Sex** | **KPS** | | **Initial**  **Stage** | | **Histology** | | **Sample Site** | | **Periop**  **Therapy** | | **Tx Lines** | | **OS (mon)** | | **PD-L1**  **(M-score^#^)** | | **CD8+ T-cell** | **CD3+ T-cell** | |
| --- | --- | --- | --- | --- | --- | --- | --- | --- | --- | --- | --- | --- | --- | --- | --- | --- | --- | --- | --- | --- | --- |
| ***Thymomas*** | | | | | | | | | | | | | | | | | | | | | |
| 1 | 56 | M | 90 | III | | B2 + B3 ^Ŧ^ | | Thymectomy | | Yes | | 3 | | 111.7 | | 100 | | 3 | | | 3 |
| 2 | 50 | M | 90 | IVA | | B3 | | Lung Wedge | | Yes | | 0 | | 23.5 | | 95 | | 3 | | | 3 |
| 3 | 33 | M | 90 | II | | B2 | | Mediastinal Mass | | Yes | | 0 | | 16.0 | | 95 | | 3 | | | 3 |
| 4 | 59 | F | 90 | IVB | | B3 | | Thymectomy | | Yes | | 0 | | 86.6 | | 90 | | 1 | | | 1 |
| 5 | 36 | F | 90 | IVA | | B2 + B3Ŧ | | Thymectomy | | Yes | | 0 | | 17.8 | | 90 | | 3 | | | N/A* |
| 6 | 42 | M | 90 | II | | B3 | | Thymectomy | | No | | 0 | | 99.3 | | 90 | | 2 | | | 2 |
| 7 | 83 | F | 90 | III | | B3 | | Mediastinal Mass | | No | | 0 | | 8.1 | | 75 | | 2 | | | 1 |
| 8 | 56 | M | 80 | IVB | | B3 | | Lung resection | | Yes | | 4 | | 140.4 | | 70 | | 3 | | | 3 |
| 9 | 74 | M | 100 | II | | B3 | | Thymectomy | | Yes | | 0 | | 63.4 | | 65 | | 2 | | | 2 |
| 10 | 45 | F | 90 | III | | B3 | | Pleura | | Yes | | 4 | | 62.6 | | 60 | | 2 | | | 1 |
| 11 | 69 | F | 80 | IVA | | B3 | | Mediastinal Mass | | Yes | | 1 | | 43.2 | | 30 | | 1 | | | 0 |
| 12 | 58 | F | 80 | IVA | | B3 | | Mediastinal Mass | | Yes | | NA* | | 11.6 | | 0 | | 1 | | | 1 |
| ***Thymic Carcinomas*** | | | | | | | | | | | | | | | | | | | | | |
| 1 | 44 | M | 80 | II | | Squamous | | Lingula | | Yes | | 1 | | 79.2 | | 90 | | 2 | | | 1 |
| 2 | 67 | F | 90 | I | | Squamous | | Thymectomy | | No | | 0 | | 72.6 | | 80 | | 3 | | | 3 |
| 3 | 72 | M | 90 | IVA | | Squamous | | Mediastinal Mass | | Yes | | 1 | | 34.4 | | 60 | | 3 | | | 3 |
| 4 | 56 | M | 80 | III | | Squamous | | Thymectomy | | Yes | | 0 | | 176.5 | | 25 | | 2 | | | 3 |
| 5 | 58 | M | 90 | IVB | | Squamous | | Mediastinal Mass | | Yes | | NA* | | 29.0 | | 20 | | 2 | | | 2 |
| 6 | 56 | M | 80 | IVB | | Squamous | | Thymectomy | | Yes | | 4 | | 70.4 | | 15 | | 2 | | | 2 |
| 7 | 79 | F | 90 | I | | Squamous | | Thymectomy | | No | | 0 | | 14.9 | | 9 | | 3 | | | 3 |
| 8 | 68 | F | 80 | III | | Squamous | | Thymectomy | | Yes | | 0 | | 80.3 | | 1 | | 2 | | | 2 |
| 9 | 70 | F | 80 | IVA | | Basaloid | | Mediastinal Mass | | Yes | | 1 | | 39.1 | | 0 | | 1 | | | 1 |
| 10 | 70 | M | 70 | III | | Squamous | | Chest wall mass | | Yes | | 2 | | 22.9 | | 0 | | 1 | | | 1 |
| 11 | 48 | M | 90 | IVB | | Squamous | | Mediastinal Mass | | Yes | | 3 | | 41.2 | | 0 | | 1 | | | 1 |

*NA= not available, #M-score= membranous tumor cell staining with PD-L1 antibody clone E1L3N, where 100 tumor cells are scored, and ≥25 positive cells represents PD-L1 positivity, mon=months, basaloid= this case is a basaloid thymic carcinoma, , Ŧ= samples present combined WHO Type B2+B3 histology where the tumor was found to be heterogeneous and tumors listed contained areas of borht B2 and B3 histology Tx= treatment.
