# Supplementary material for: Expression of PD-L1 and other immunotherapeutic targets in thymic epithelial tumors
Source: PLoS One. 2017 Aug 3;12(8):e0182665. doi: 10.1371/journal.pone.0182665 (PMC5542609; doi:10.1371/journal.pone.0182665)
Supplement: S2 Table — (DOCX) [file pone.0182665.s002.docx]

**S2 Table: Pairwise Association of Co-Stimulatory and Co-Inhibitory Immune Checkpoint Molecules with PD-L1 positivity and TILs**

|  | **CD137** | **CD8** | **CD3** | **CTLA4** | **ICOS** | **GITR** | **TIM3** | **PD1** |
| --- | --- | --- | --- | --- | --- | --- | --- | --- |
|  | p-value | p-value | p-value | p-value | p-value | p-value | p-value | p-value |
| **CD8** | 0.01 |  |  |  |  |  |  |  |
| **CD3** | 0.02 | 0.001 |  |  |  |  |  |  |
| **CTLA4** | 0.008 | 0.069 | 0.192 |  |  |  |  |  |
| **ICOS** | 0.03 | 0.64 | 0.08 | 0.012 |  |  |  |  |
| **GITR** | 0.084 | 0.09 | 0.08 | 0.22 | 0.1 |  |  |  |
| **TIM3** | 0.135 | 0.089 | 0.116 | 0.155 | 1 | 1 |  |  |
| **PD1** | 0.08 | 0.323 | 0.367 | 0.22 | 0.67 | 0.043 | 1 |  |
| **PD-L1** | 1 | 0.131 | 0.662 | 0.4 | 1 | 1 | 0.297 | 1 |

*Matrix of p-values from pairwise association analysis. Markers were compared as binary values, M-score: defined as >25 of 100 cells with positive membranous staining of PD-L1, all other markers evaluated by immunohistochemistry, where 0/1= low positive T cell staining, 2/ 3+= moderate-high positive T cell staining.
